# Supplementary material for: Aversive stimulus-tuned responses in the CA1 of the dorsal hippocampus
Source: Nat Commun. 2023 Oct 27;14:6841. doi: 10.1038/s41467-023-42611-w (PMC10611787; doi:10.1038/s41467-023-42611-w)
Supplement: Supplementary file 1 — Supplementary Information [file 41467_2023_42611_MOESM1_ESM.pdf]

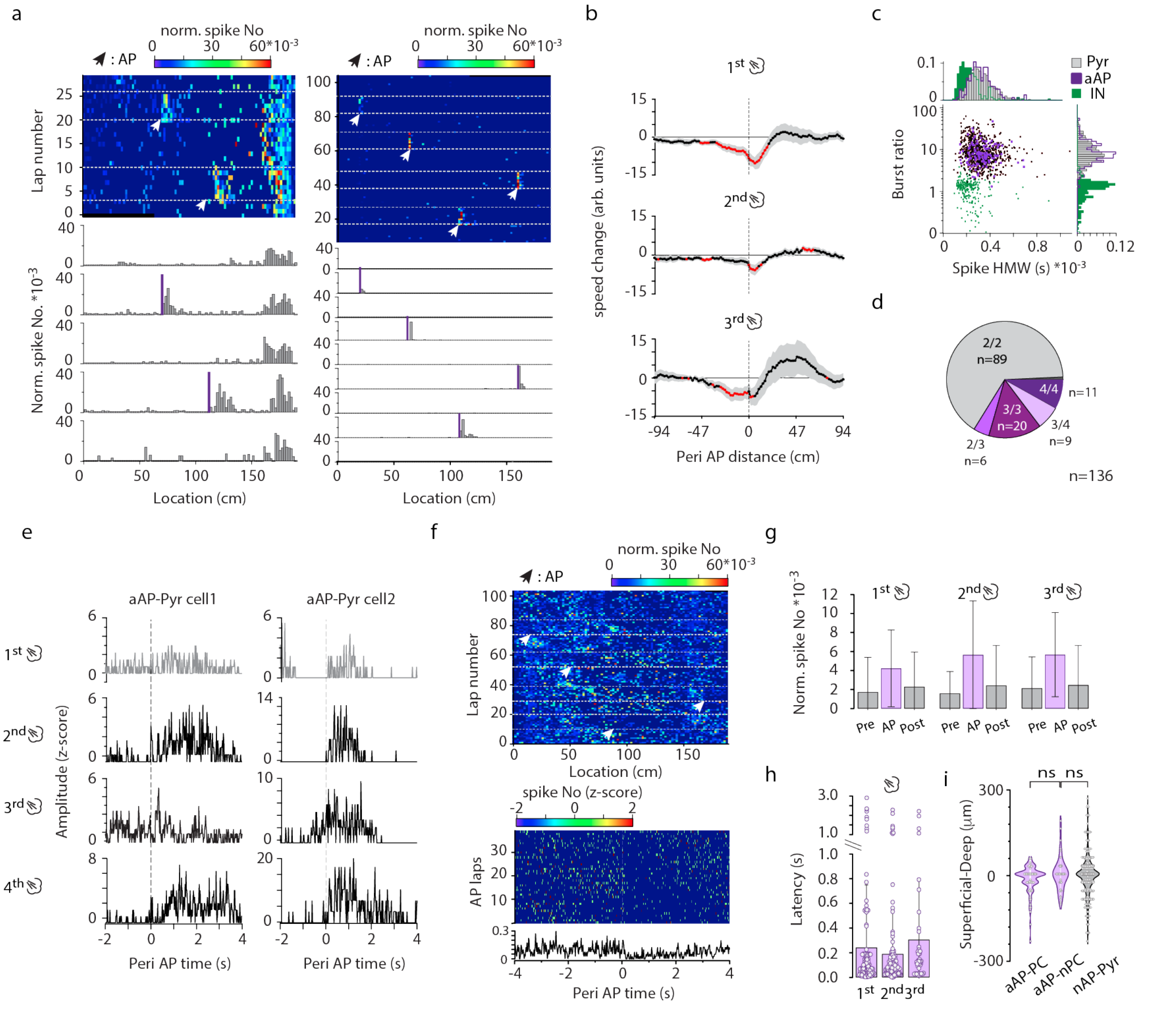

Supplementary Figure 1

AP-triggered responses (**a**) Representative location – lap number raster of a mixed tuning aAP-PC (left) and an aAP-nPC pyramidal cell (right). Note very short latency, temporally focused activation of the aAP-nPC. White arrowheads mark air puff locations. Light grey dashed lines separate consecutive no stimulation and air puff (2, 4) epochs. Mean tuning curves in control and AP epochs are plotted below the location-trial raster. (**b**) Differences in speed around the AP stimulation between no stimulation and air puff epochs, calculated in the 1st, 2nd and 3rd AP epochs. Red indicates significant difference, shaded area corresponds to s.e.m, for testing significance Wilcoxon signed-rank test was used, $p<0.05$. (**c**) Burst ratio as the function of spike width at the half of maximum. Black dots represent putative pyramidal cells (Pyr), green dots represent putative interneurons (IN) and violet dots represent AP activated pyramidal cells (aAP). Along the axes are the marginal distributions. (**d**) Partitioning of aAP-Pyr cells based on the number of AP epochs in which significant activation could be detected per the total number of AP epochs delivered in the respective session. (**e**) Peri-air puff firing histograms of two example aAP-Pyr cells in each air puff epoch (4 air puff locations). Both neurons increased activity to variable extent but elevation exceeded significance only in 3/4 epochs. (**f**) Location – lap number raster (upper) and peri-air puff firing histogram (lower) of an iAP-nPC. (**g**) Summarized data showing the normalized spike number before (Pre), during (AP) and after (Post) air puff responses at the first, second and third AP locations (2-way repeated measures ANOVA: not significant location factor $F(2,106)=1.21$, significant main effect of stimulus

$F(2,106)=36.7$, $p=7.9*10^{-13}$). (**h**) Summarized data showing the latency of air puff responses at the first, second and third air puff locations (1st: 0.24±0.51 s; 2nd: 0.19±0.36 s; 3rd: 0.31±0.49 s, one-way ANOVA, $F(2,302)=1.18$, $p=0.31$). (**i**) Location of AP-activated place cells (aAP-PC), AP-activated non-place cells (aAP-nPC) and AP-non-affected (nAP) pyramidal cells relative to the middle of pyramidal layer (Kruskal-Wallis test, $H(2) = 2.8$, $p = 0.24$). Source data of panel c,g,h and i are provided as a Source Data file.

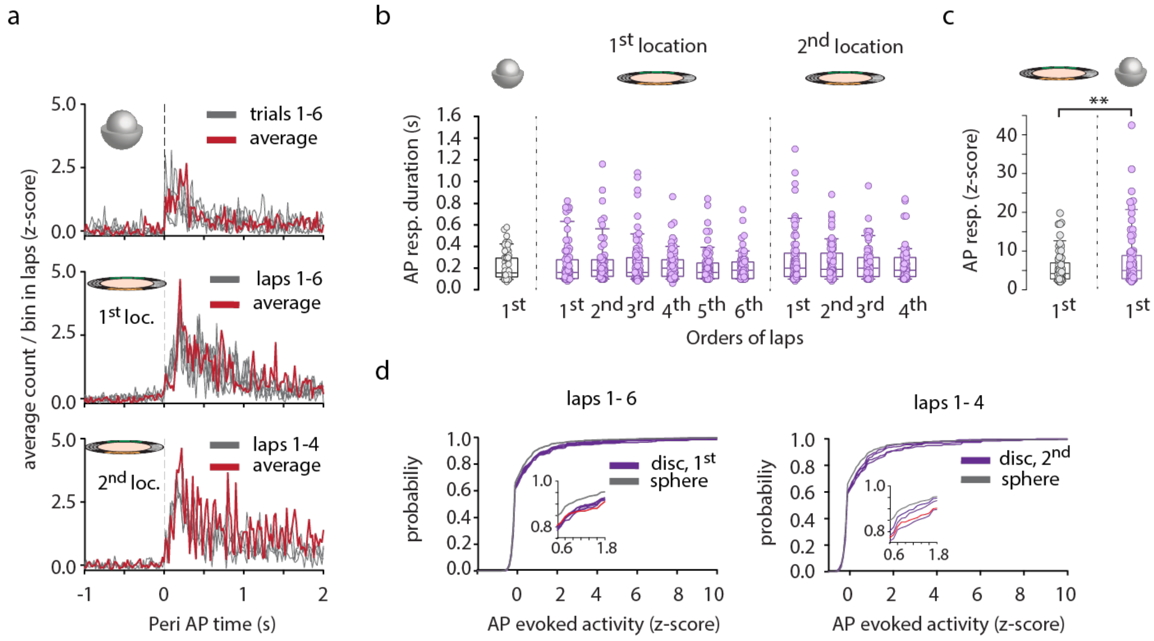

Supplementary Figure 2

There's no difference in AP-evoked responses among different laps (**a**) Spiking activity of air puff-activated putative pyramidal cells on the sphere and on the disc (1st and 2nd air puff locations). Grey lines indicate the averages of individual laps from all sessions at the disc and averages of the individual first 6 trials at the sphere. Red traces indicate the average responses in the first laps/trials. Note, that there's an increased spiking activity even at the very first trial of air puff stimulation. (**b**) Duration of air puff-evoked spiking activity in the individual laps at the first air puff location (6 laps, n= 89 aAP-Pyr cells from 5 sessions) and second air puff location (4 laps, n= 89 aAP-Pyr cells from n=5 session) and on the sphere. Circles represent units, box and whiskers correspond to median, quartile and 10-90% range. (Kruskal-Wallis test, for first location: $H(5) = 3.82$, $p = 0.57$, for second location: $H(3) = 0.81$, $p = 0.84$) (**c**) Z-scored air puff-evoked activation at the very first lap on the disc (n=57) and in the first trial on the sphere. Circles represent cells, box and whiskers correspond to median, quartile and 10-90% range. Wilcoxon signed rank test $^{**}p=0.005$ (**d**) Cumulative distribution of air puff-evoked activity in individual laps (violet) at the first location on the disc (left panel) and at the second location on the disc (right panel). Grey line represents activity on the sphere. Inset highlights the differences in the distribution of air puff-evoked activity on the disc vs sphere. The first lap on the disc (1st and 2nd location) is highlighted by red color. Source data of panel b and c are provided as a Source Data file.

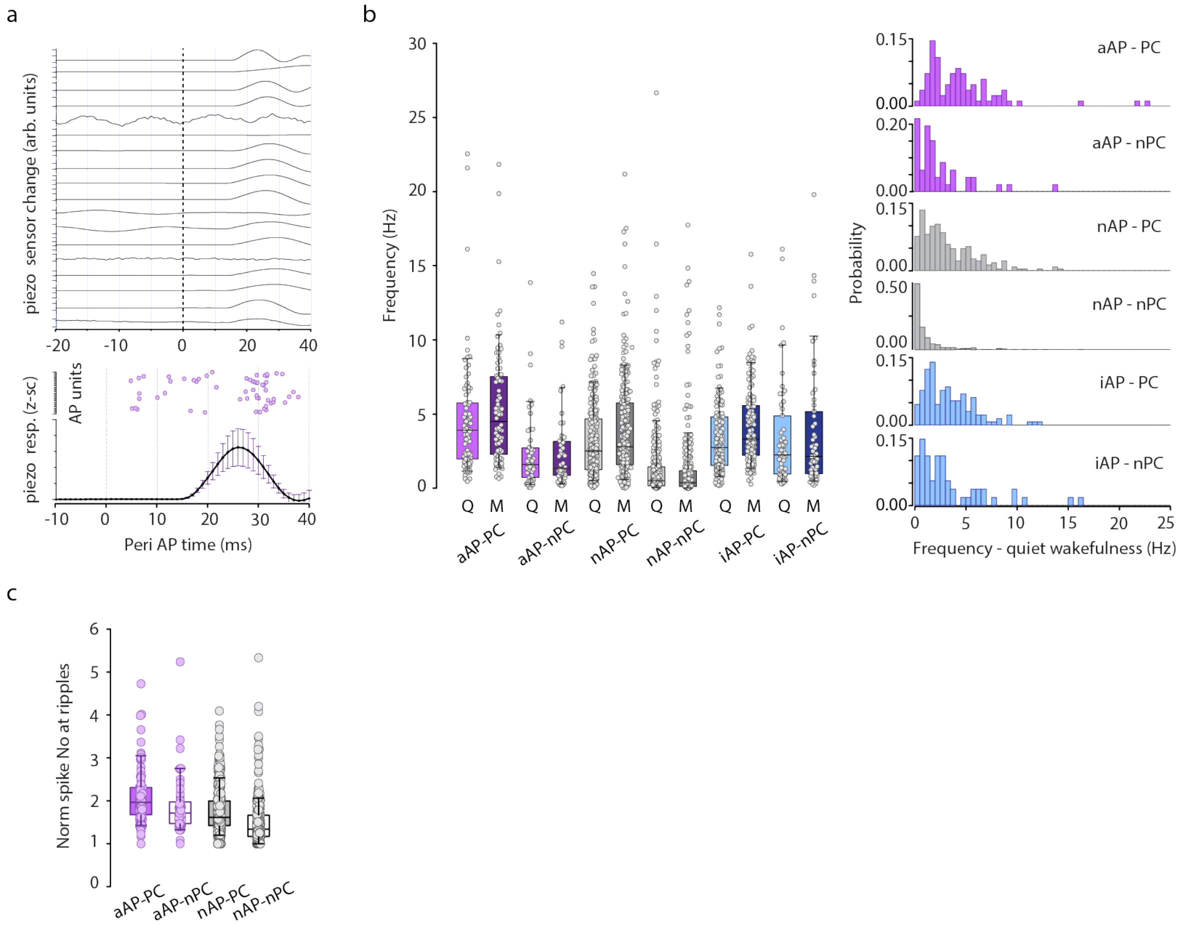

Supplementary Figure 3

Basic properties of aAP-Pyr cells and nAP-Pyr cells. (**a**) Piezo lick sensor signals around the air puff onset (n = 18 sessions). Bottom panel: average piezo sensor signal around air puff onset. Violet circles indicate the first spikes of aAP-Pyr cells. Error bars show s.e.m. (**b**) Firing frequency during quiet wakefulness (Q) and movement (darker colors, M) periods. Cells were grouped based on air puff response. aAP-PC, air puff-activated place cells; aAP-nPC, air puff-activated non-place cells; nAP-PC, air puff-non-responsive place cell; aAP-nPC, air puff-non-responsive, non-place cell; iAP-PC, air puff-inhibited place cell; iAP-nPC, air puff-inhibited non-place cells. Two-way repeated measures ANOVA: Factor A (quiet-mov) F(1,777)=76.12, p<0.0001, Factor B (cell groups) F(5,777)=21.72, p=2.17*10^{-20}, interactions F(5,777)=9, p=2.48*10^{-8}. There were significant differences with Post hoc Tukey test: p<0.05: aAP-nPC vs nAP-PC and aAP-PC vs iAP-nPC. p<0.01: aAP-nPC vs iAP-PC, nAP-nPC vs iAP-nPC, aAP-PC vs nAP-PC, nAP-nPC vs aAP-PC or nAP-PC or iAP-PC and aAP-PC vs iAP-PC. Right panel, Distribution of firing frequencies of the cell groups during quiet wakefulness. (**c**) Normalized spike number during “active” ripples (total spike number during ripples / number of ripples during which the unit emitted at least one spike) of air puff-activated place cells (aAP-PC), air puff-activated non-place cells (aAP-nPC), air puff-non-responsive place cells (nAP-PC) and air puff-non-responsive non-place cells (nAP-nPC). Kruskal-Wallis test H(3)=113.7, p<0.001, Dunn-Holland-Wolfe post-hoc test: aAP-PC vs nAP-PC or nAP-nPC p<0.05, aAP-nPC vs nAP-nPC p<0.05, nAP-PC vs nAP-nPC p<0.05. On panels b and c, box and whiskers correspond to median, quartile and 10-90% range. Source data of panel b and c are provided as a Source Data file.

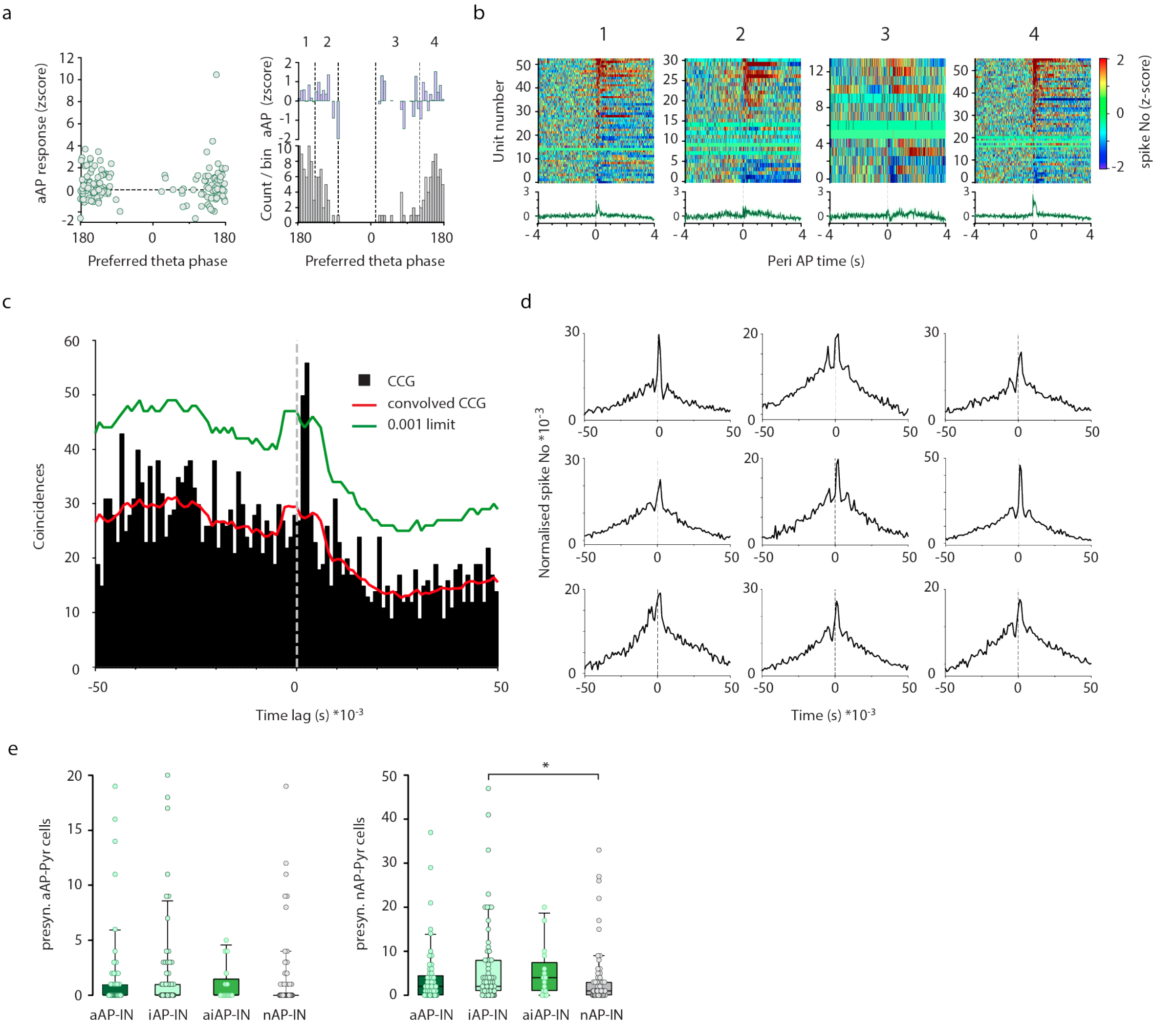

Supplementary Figure 4

Theta phase and monosynaptic connections of recorded interneurons. (**a**) Z-scored air puff-activated responses as the function of the preferred theta phase of the interneuron. Dashed line indicates zero level (no effect). Right panel: mean z-scored air puff responses (upper panel) in 20^{0} phase bins and corresponding phase histogram (lower panel) of interneurons. Dashed lines indicate the four groups of interneurons based on theta preference. (**b**) Peri-air puff firing histograms of the 4 phase groups of interneurons. Group numbers are labeled at the top of each panel. Averaged peri-air puff firing histograms of interneurons are shown below the individual histograms, shaded areas correspond to s.e.m. (**c**) A representative pyramidal cell – interneuron crosscorrelogram with a significant peak indicating a putative monosynaptic connection. Red line: Gaussian-convolved crosscorrelogram; green line: significance level ($p=0.001$) determined based on Poisson distribution. (**d**) Representative crosscorrelograms between air puff-activated pyramidal cells and a target air puff-activated putative interneuron (see Fig. 2i). (**e**) Average number of aAP-Pyr (left panel) or nAP-Pyr (right panel) cells among the presynaptic partners of interneurons with different air puff response types. Circles represent cells, box and whiskers correspond to median, quartile and 10-90% range. Kruskal-Wallis test, aAP-Pyr cells: $H(3) = 4.4$, $p = 0.22$, nAP-Pyr cells: $H(3) = 14.54$, $p = 0.002$. Dunn-Holland-Wolfe post hoc test aAP-IN vs aiAP-IN *$p < 0.05$. Source data of panel a and e are provided as a Source Data file.

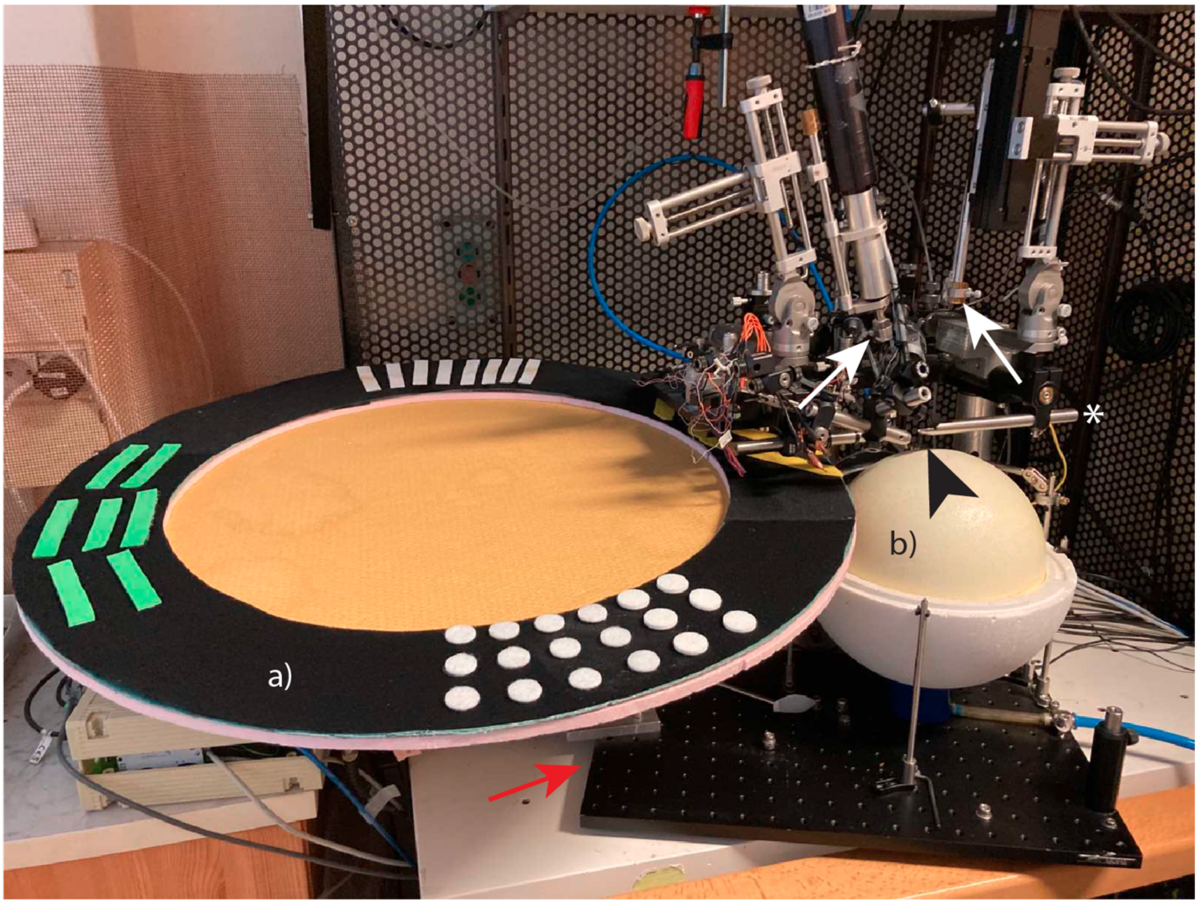

Supplementary Figure 5

Photo of the recording setup. a) and b) represent the disc and sphere, respectively. The black arrowhead points to the location of the mouse's head, which is restrained through the headpost fitted into the metal rods on the right and left sides, labeled with asterisks. Motorized micromanipulators are fixed onto a stereotaxis arm on both the left and right sides, with white arrows indicating the probeholders. The sliding mechanism that allows switching between the sphere and disc, is located underneath the breadboard, red arrow shows to the location.

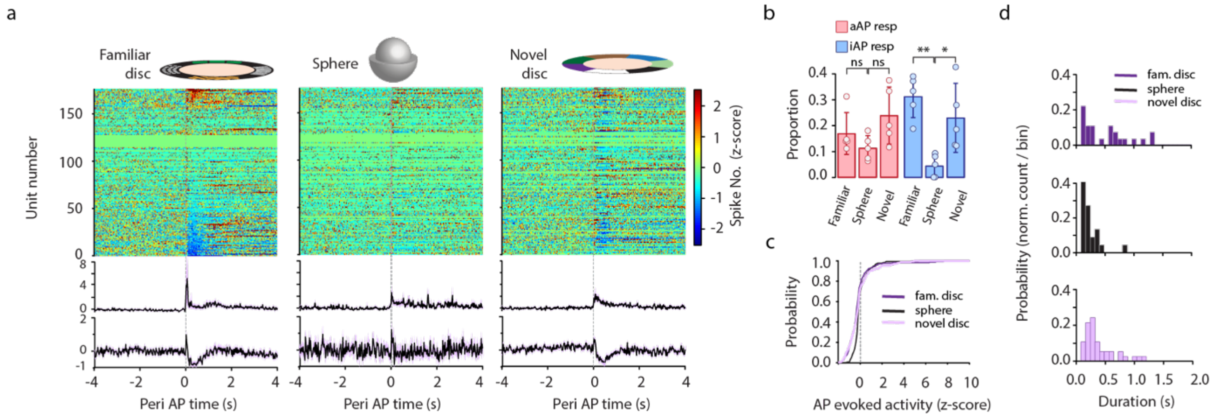

Supplementary Figure 6

Air puff elicited pyramidal cell response is strongly modulated by spatial information in the environment. (**a**) Peri air puff firing histograms of putative pyramidal cells (n=177 from 5 sessions) on familiar disc (left panel), sphere (middle panel) and novel disc (right panel). Mean peri-air puff firing histograms of significantly activated and suppressed pyramidal cells are shown below the individual histograms. Z-scored response magnitude of putative pyramidal cells with facilitated responses: familiar disc: 8.6 ± 19.1, sphere: 5.1 ± 2.5, novel disc: 4.7 ± 3.0, one-way-ANOVA $F(2,83)=1.1$, $p = 0.33$. Shaded areas correspond to s.e.m. (**b**) Summarized data of the proportion of air puff-activated putative pyramidal cells (5 session) on familiar disc: mean and SD values are 0.17 ± 0.08, sphere: 0.11 ± 0.05 and novel disc: 0.24 ± 0.11; one-way-ANOVA $F(2,12)=2.8$, $p=0.1$. Suppressed pyramidal cells on familiar disc: 0.31 ± 0.08, sphere: 0.04 ± 0.04, novel disc: 0.23 ± 0.13; one-way-ANOVA $F(2,12)=10.7$, $p=0.002$, Tukey's post-hoc: sphere vs familiar disc: $^{**}p=0.002$, sphere vs novel disc: $^{*}p=0.02$. (**c**) Cumulative distribution of air puff-responding putative pyramidal cell response magnitudes on familiar disc (violet line), sphere (black) and novel disc (light violet). Kolmogorov-Smirnov test: n=177 pyramidal cells; familiar disc vs. sphere: $^{**}p=3x10^{-6}$, sphere vs. novel disc: $^{**}p=7x10^{-5}$. (**d**) Distribution of air puff-response durations of putative pyramidal cells on familiar disc (violet, upper panel), sphere (black, middle panel) and novel disc (light violet, lower panel): Mean and SD values are: fam. disc: 0.73 ± 0.77 s, sphere: 0.24 ± 0.17 s, novel disc: 0.40 ± 0.28 s; one-way-ANOVA $F(2,83)=7.0$, $p = 0.0016$. Tukey's post-hoc: sphere vs familiar disc: $p=0.002$, sphere vs novel disc: $p = 0.43$. For visualization purposes time axis was truncated at 2 s. Source data of panel b are provided as a Source Data file.

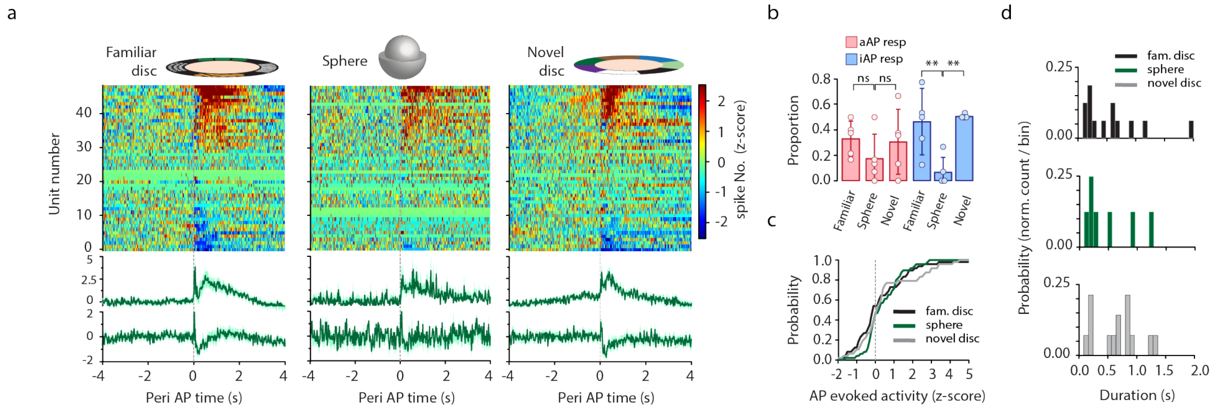

Supplementary Figure 7

Air puff elicited interneuron response is strongly modulated by spatial information in the environment. (**a**) Peri air puff firing histograms of putative interneurons (n=49 from 5 sessions) on familiar disc (left panel), sphere (middle panel) and novel disc (right panel), units are sorted individually. Mean peri-air puff firing histograms of significantly activated and suppressed interneurons are shown below the individual histograms. Mean and SD values of the z-scored response magnitude of interneurons with facilitated responses: familiar disc: 5.0 ± 2.3, sphere: 5.4 ± 1.9, novel disc: 4.4 ± 1.1, one-way-ANOVA F(2,35)=0.85, p = 0.44. Shaded areas correspond to s.e.m. (**b**) Summarized data of the proportion of air puff-activated putative interneurons (5 session) on familiar disc: 0.33 ± 0.14, sphere: 0.17 ± 0.2 and novel disc: 0.31 ± 0.25; one-way-ANOVA F(2,12)=0.88, p = 0.44. Suppressed interneurons on familiar disc: 0.47 ± 0.26, sphere: 0.07 ± 0.11, novel disc: 0.51 ± 0.02; one-way-ANOVA F(2,12)=10.7, p=0.002, Tukey's post-hoc: sphere vs familiar disc: **p=0.007, sphere vs novel disc: **p = 0.003. (**c**) Cumulative distribution of putative interneuron response magnitudes on familiar disc (black line), sphere (green) and novel disc (grey). Kolmogorov-Smirnov test: n=49 interneurons; familiar disc vs. sphere: p=0.09, sphere vs. novel disc: p=0.23. (**d**) Distribution of air puff-response durations of putative interneurons on familiar disc (0.85 ± 0.79 s, n=16, black, upper panel), sphere (0.70 ± 0.7 s, n=8, green, middle panel) and novel disc (0.65 ± 0.38 s, n=14, grey, lower panel), one-way-ANOVA F(2,35)=0.40, p = 0.68. Source data of panel b are provided as a Source Data file.

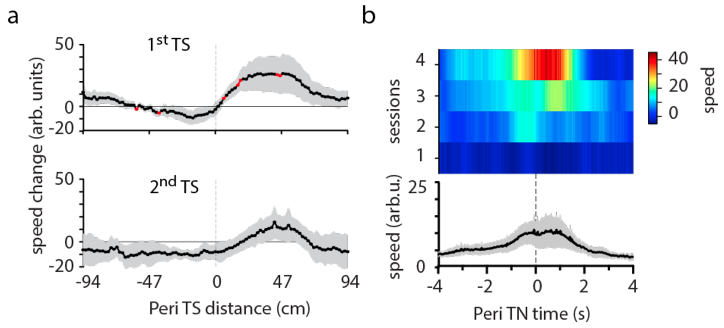

Supplementary Figure 8

Behavioral response to aversive tail-shock and neutral tone. (**a**) Difference of control and tail shock-triggered speed calculated for the 1st and 2nd TS epochs. Red dots correspond to statistically different control vs. TS-triggered speed, shaded area corresponds to s.e.m, for testing significance Wilcoxon signed-rank test was used, $p<0.05$. (**b**) Auditory stimulus-triggered time histogram of speed values. Upper panel: rows of color-scaled matrix show mean tone onset-triggered speed in individual sessions. Lower panel: all session average of tone onset-triggered speed. Color bar: speed (arb. unit).

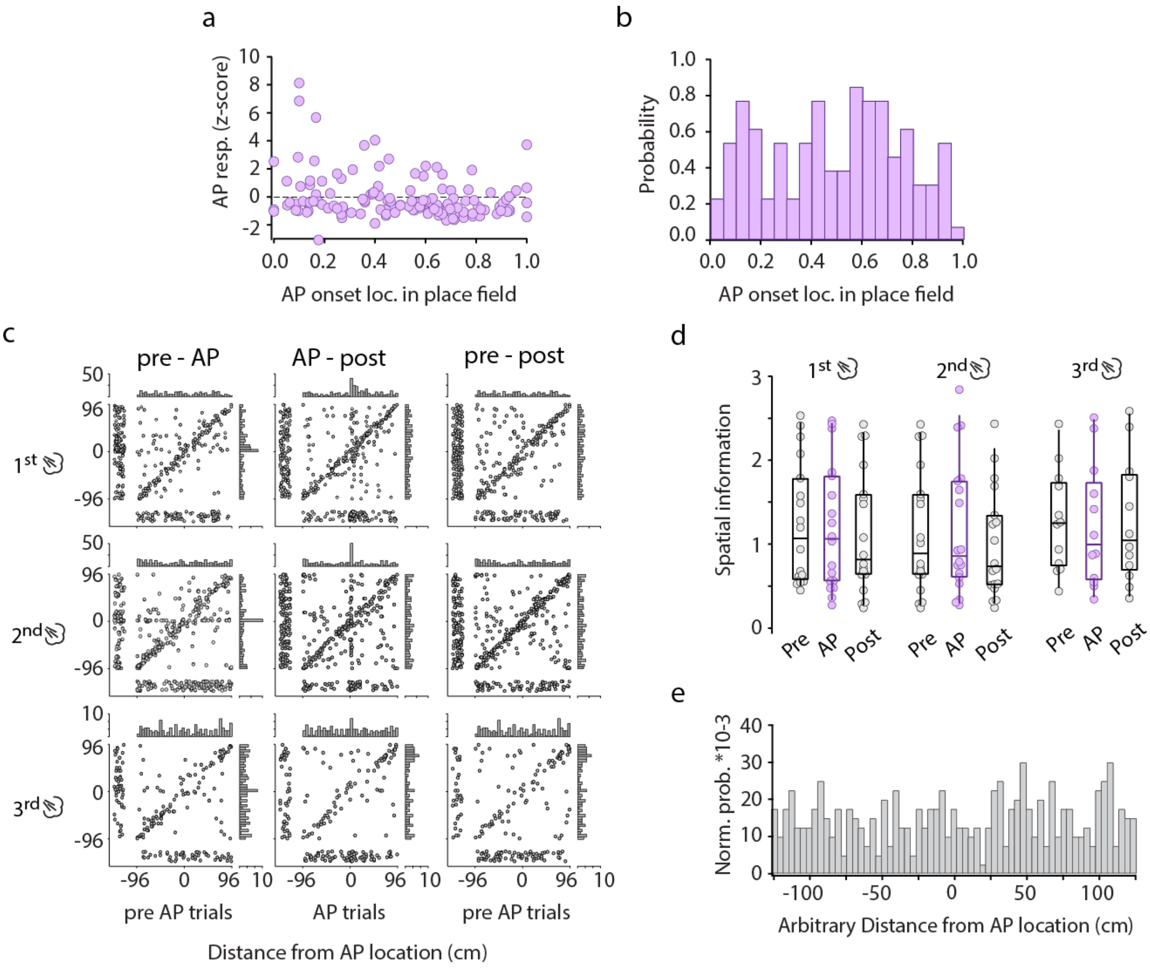

Supplementary Figure 9

Place cells' remapping by aversive stimulation (**a**) Z-scored air puff response of putative pyramidal cells as a function of normalized air puff location in the place field. Circles represent individual cells (n=133, same cells as in Figure 6a). (**b**) Distribution of normalized air puff onset location in place field. (**c**) Place field distances from air puff location during air puff epochs (AP) versus pre air puff epochs (pre, first column), during air puff epochs (AP) versus post air puff epochs (post, second column) and during post air puff epochs (post) versus pre air puff epochs (pre, third column). Rows correspond to different air puff locations. Circles represent place field centers. (**d**) Spatial information (calculated using equation 1, see methods) before the air puff stimulus (pre), during the air puff epochs (AP) and after the air puff stimulus (Post) at the first- second and third air puff locations. Two-way repeated measures ANOVA epoch factor $F(2,18)=0.11$, $p=0.89$, location factor $F(2,18)=0.63$, $p=0.54$. (**e**) Peri-air puff distribution of stable place fields in pre versus post air puff epochs (Fig. 6c, place field distribution along the diagonal). Source data of panel a,c and d are provided as a Source Data file.

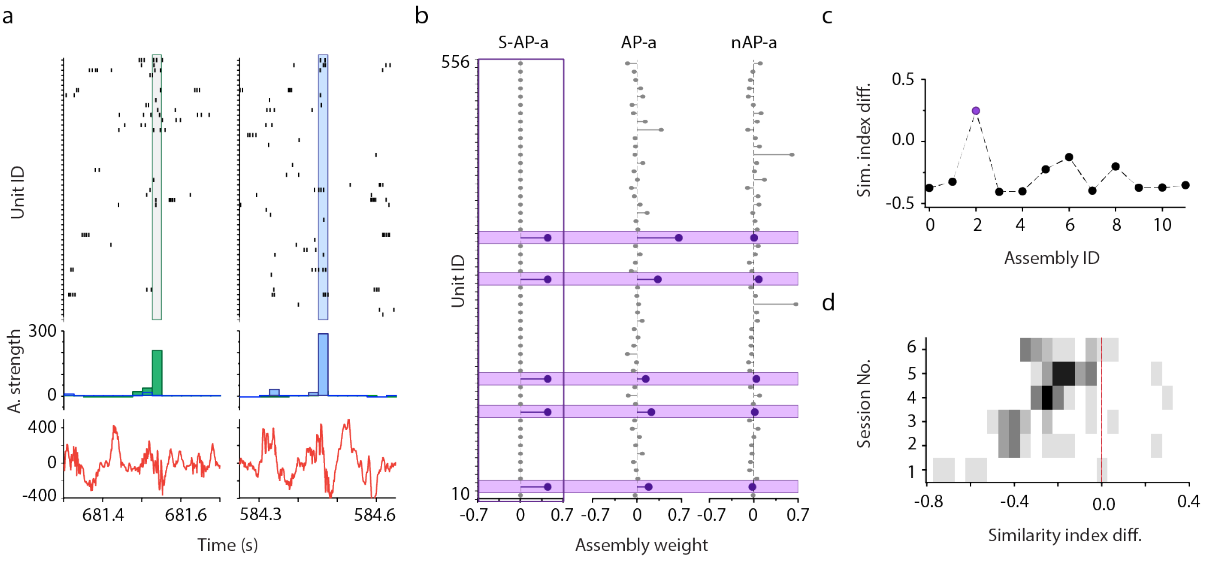

Supplementary Figure 10

Detection of air puff coupled assemblies. (**a**) Two representative assemblies (green and blue) are shown on the top. Raster plot indicates unit activity. Colored rectangles highlight units belonging to the assemblies. Middle: assembly reactivation strength. Bottom: local field potential showing ripple activity during assembly reactivation. (**b**) Schematic indicating the weights of individual units for a synthetic air puff assembly (S-AP-a, left), the air puff assembly (AP-a, middle) detected based on similarity index distribution and a different, non-air puff assemblies (nAP-a). Violet shadows highlight the air puff cells. (**c**) Similarity index differences calculated from the bootstrapped threshold value for11 assemblies in a sample session. One assembly was defined as air puff assembly (violet) based on the similarity index differences. (**d**) Similarity index distributions from threshold values for all assemblies of six sessions. Red line represents threshold for significance. Note the air puff assemblies on the right side of the vertical red line. Source data of panel c are provided as a Source Data file.

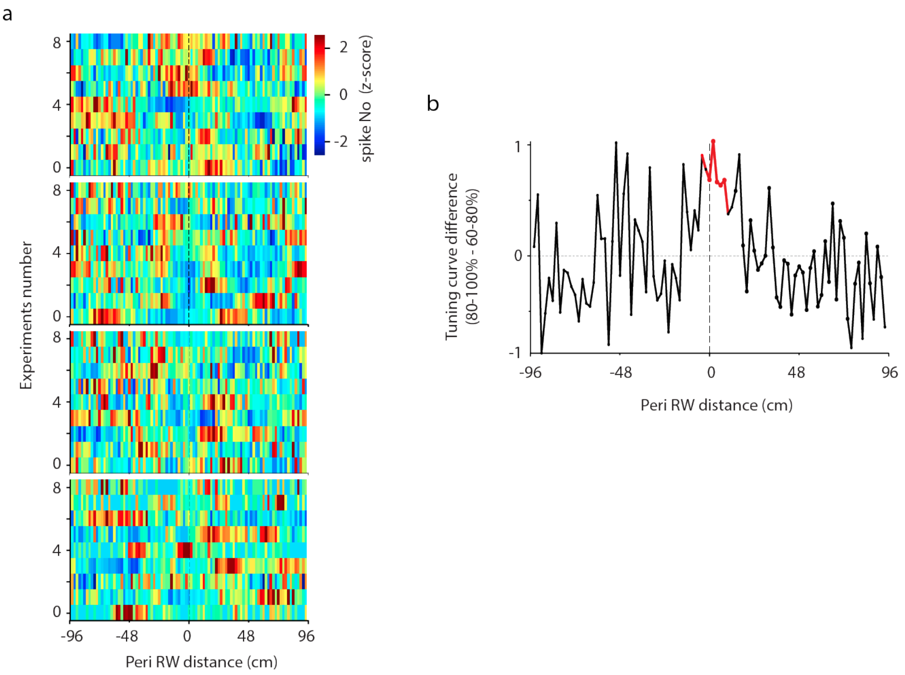

Supplementary Figure 11

Reward-coding theta cycles are dominated by place cells with reward-proximal place fields. (**a**) Peri-reward average tuning curves of place cells in activity quintiles calculated by sorting place cells based on their activity in reward-coding theta cycles in a 3 s window following air puff stimulus; uppermost plot: 80%-100% activity quintile, lowermost: 20%-40% activity quintile. (**b**) Mean difference between tuning curves in the highest and second highest activity quintiles. Red marks significant difference between tuning curves (Wilcoxon Mann-Whitney test, n=8 average per session tuning curves, *$p<0.05$).
