# Supplementary material for: Aversive stimulus-tuned responses in the CA1 of the dorsal hippocampus
Source: Nat Commun. 2023 Oct 27;14:6841. doi: 10.1038/s41467-023-42611-w (PMC10611787; doi:10.1038/s41467-023-42611-w)
Supplement: Supplementary file 3 — Reporting Summary [file 41467_2023_42611_MOESM3_ESM.pdf]

nature portfolio

Corresponding author(s): Albert M Barth, Viktor Varga

Last updated by author(s): Sep 18, 2023

Reporting Summary

Nature Portfolio wishes to improve the reproducibility of the work that we publish. This form provides structure for consistency and transparency in reporting. For further information on Nature Portfolio policies, see our Editorial Policies and the Editorial Policy Checklist.

Statistics

For all statistical analyses, confirm that the following items are present in the figure legend, table legend, main text, or Methods section.

n/a	Confirmed	
☐	☒	The exact sample size (*n*) for each experimental group/condition, given as a discrete number and unit of measurement
☐	☒	A statement on whether measurements were taken from distinct samples or whether the same sample was measured repeatedly
☐	☒	The statistical test(s) used AND whether they are one- or two-sided<br>*Only common tests should be described solely by name; describe more complex techniques in the Methods section.*
☐	☒	A description of all covariates tested
☐	☒	A description of any assumptions or corrections, such as tests of normality and adjustment for multiple comparisons
☐	☒	A full description of the statistical parameters including central tendency (e.g. means) or other basic estimates (e.g. regression coefficient) AND variation (e.g. standard deviation) or associated estimates of uncertainty (e.g. confidence intervals)
☐	☒	For null hypothesis testing, the test statistic (e.g. F, t, r) with confidence intervals, effect sizes, degrees of freedom and P value noted<br>*Give P values as exact values whenever suitable.*
☐	☒	For Bayesian analysis, information on the choice of priors and Markov chain Monte Carlo settings
☒	☐	For hierarchical and complex designs, identification of the appropriate level for tests and full reporting of outcomes
☐	☒	Estimates of effect sizes (e.g. Cohen's *d*, Pearson's *r*), indicating how they were calculated

Our web collection on statistics for biologists contains articles on many of the points above.

Software and code

Policy information about availability of computer code

Data collection	Data acquisition software: Open Ephys 0.4.4.1
Data analysis	Spike sorting software: Spyking Circus and Phy2. Data were analyzed using custom code in Igor Pro 8 and 9, for assembly analysis Python and Igor Pro 9 were used, for 2-way repeated measures ANOVA Microsoft Excel was used. Custom Igor Pro codes were deposited at Zenodo on the following link: DOI 10.5281/zenodo.8343626

For manuscripts utilizing custom algorithms or software that are central to the research but not yet described in published literature, software must be made available to editors and reviewers. We strongly encourage code deposition in a community repository (e.g. GitHub). See the Nature Portfolio guidelines for submitting code & software for further information.

Data

Policy information about availability of data

All manuscripts must include a data availability statement. This statement should provide the following information, where applicable:

- Accession codes, unique identifiers, or web links for publicly available datasets
- A description of any restrictions on data availability
- For clinical datasets or third party data, please ensure that the statement adheres to our policy

Raw data (electrophysiological recordings) were deposited at Zenodo. Accession codes are as follows:
DOI 10.5281/zenodo.8339978

DOI 10.5281/zenodo.8341204
DOI 10.5281/zenodo.8341254
DOI 10.5281/zenodo.8341258
DOI 10.5281/zenodo.8341264
DOI 10.5281/zenodo.8341268
DOI 10.5281/zenodo.8341280
DOI 10.5281/zenodo.8343580
DOI 10.5281/zenodo.8343582
DOI 10.5281/zenodo.8343572
DOI 10.5281/zenodo.8343509
DOI 10.5281/zenodo.8343558

Research involving human participants, their data, or biological material

Policy information about studies with human participants or human data. See also policy information about sex, gender (identity/presentation), and sexual orientation and race, ethnicity and racism.

Reporting on sex and gender	N/A
Reporting on race, ethnicity, or other socially relevant groupings	N/A
Population characteristics	N/A
Recruitment	N/A
Ethics oversight	N/A

Note that full information on the approval of the study protocol must also be provided in the manuscript.

Field-specific reporting

Please select the one below that is the best fit for your research. If you are not sure, read the appropriate sections before making your selection.

☒ Life sciences ☐ Behavioural & social sciences ☐ Ecological, evolutionary & environmental sciences

For a reference copy of the document with all sections, see nature.com/documents/nr-reporting-summary-flat.pdf

Life sciences study design

All studies must disclose on these points even when the disclosure is negative.

Sample size	The following factors were considered when determining the number of animals used: i) number of neurons needed to be collected for detecting at least one aversive stimulus-tuned neuron based on the average proportion of these cells. We were able to estimate this proportion only after the initial experiments therefore a prior estimation could not be carried out. ii) The number of separable units differs significantly from animal to animal (from ~ 30 to ~ 140 in our case) therefore we attempted to lower the chance that one exceptionally high yielding animal dominate the sample by increasing both the number of simultaneously recorded neurons (deploying up to 3 high -128-channel count probes) and the number of animals. Because predicting per animal yield is not possible, we updated the number of experiments to be done based on the yield of preceding experiments. Notably, the final number of animals used falls within the range reported in the literature (e.g. Modi et al., 2023, eLife; Peyrache et al., 2015, Nat.Neurosci.; Fischler-Ruiz et al., 2021, Neuron).
Data exclusions	In general, data were not excluded from the analyses. During spike sorting noisy clusters were discarded. If the assembly analysis failed to identify significant assemblies (e.g. the weights of neurons did not reached significance) the respected session was excluded from assembly analysis. Likewise, sessions with high decoding error were excluded from position estimation.
Replication	The same experimenter ran all experiments, but these could be separated by several weeks and other types of experiments: animals included in this study were measure in three major groups. Additionally, two mouse strains were used in these experiments and we could not detect deviation in any of the groups (e.g. no detectable response to aversive stimuli). For details on the animals with animal codes and main characteristics of the experiments could be found in Table 1.
Randomization	We did not apply any treatment in these experiments therefore animals were not separated into treatment groups. While the structure of the experiments were different in a subset of animals, the basic recording conditions were unaltered: neuronal response to aversive stimuli was registered in two recording conditions (on a spherical and on a circular treadmill) and the order of these conditions was changed (spherical - circular - spherical in one subset and circular - spherical - new circular in the other subset). The experimenter decided whether a given animal was measured according to the first or to the second scenario without prior randomization. In order to test the significance of described phenomena, we also applied Monte Carlo analysis and real data was tested against a shuffled data set. This latter analysis was applied for testing the detection of aversive stimulus-locked assemblies and the shift of decoded location from the current to the reward zone.
Blinding	There were no treatment groups in this study, therefore blinding was not applicable. However the analysis was unbiased regarding the

Blinding	described phenomena: a global significance threshold based on the distribution of firing rate values was applied for detecting aversive stimulus-tuned responses and it was memoryless i.e. the outcome of the analysis of a given animal was not influenced by results of prior animals. Additionally, multiple experiments were analyzed at once by batch processing without user intervention (and any preselection).

Reporting for specific materials, systems and methods

We require information from authors about some types of materials, experimental systems and methods used in many studies. Here, indicate whether each material, system or method listed is relevant to your study. If you are not sure if a list item applies to your research, read the appropriate section before selecting a response.

Materials & experimental systems

n/a	Involved in the study	
☒	☐	Antibodies
☒	☐	Eukaryotic cell lines
☒	☐	Palaeontology and archaeology
☐	☒	Animals and other organisms
☒	☐	Clinical data
☒	☐	Dual use research of concern
☒	☐	Plants

Methods

n/a	Involved in the study	
☒	☐	ChIP-seq
☒	☐	Flow cytometry
☒	☐	MRI-based neuroimaging

Animals and other research organisms

Policy information about studies involving animals; ARRIVE guidelines recommended for reporting animal research, and Sex and Gender in Research

Laboratory animals	Mouse, vGat-ires-Cre 8-12 weeks, vGlut3-ires-cre 8-12 weeks. Housing conditions are described in the manuscript.
Wild animals	The study did not involve wild animals.
Reporting on sex	Just male mice were used in the study but sex was not considered in study design. We do believe that the result is sex independent.
Field-collected samples	Study did not involve samples collected from the field.
Ethics oversight	Experiments were approved by the Animal Care and Use Committee of the Institute of Experimental Medicine and the Committee for Scientific Ethics of Animal Research of the National Food Chain Safety Office under the project number PE/EA/200-2/2020 and were performed according to the 2010/63/EU Directive of the EU Council.

Note that full information on the approval of the study protocol must also be provided in the manuscript.
